# Insulin-like growth factor binding protein 3 promoter variant (rs2854744) is associated with nonalcoholic fatty liver disease

**DOI:** 10.20945/2359-4292-2023-0017

**Published:** 2023-11-10

**Authors:** Touraj Mahmoudi, Shadi Nouri, Fatemeh Zarei, Zeinab Nourmohammadi Najafabadi, Maryam Sanei, Shiva Sayedsalehi, Gholamreza Rezamand, Asadollah Asadi, Reza Dabiri, Hossein Nobakht, Hamid Farahani, Seidamir Pasha Tabaeian, Mohammad Reza Zali

**Affiliations:** 1 Shahid Beheshti University Research Institute for Gastroenterology and Liver Diseases Gastroenterology and Liver Diseases Research Center Tehran Iran Gastroenterology and Liver Diseases Research Center, Research Institute for Gastroenterology and Liver Diseases, Shahid Beheshti University of Medical Sciences, Tehran, Iran; 2 Arak University of Medical Sciences School of Medicine Department of Radiology Arak Iran Department of Radiology, School of Medicine, Arak University of Medical Sciences, Arak, Iran; 3 Università degli Studi di Milano Department of Pharmaceutical Sciences Milan Italy Department of Pharmaceutical Sciences, Università degli Studi di Milano, Milan, Italy; 4 Isfahan University of Medical Sciences School of Dentistry Isfahan Iran School of Dentistry, Isfahan University of Medical Sciences, Isfahan, Iran; 5 Islamic Azad University Mashhad Branch Faculty of Medicine Mashhad Iran Faculty of Medicine, Mashhad Branch, Islamic Azad University, Mashhad, Iran; 6 Iran University of Medical Sciences School of Medicine Department of Internal Medicine Tehran Iran Department of Internal Medicine, School of Medicine, Iran University of Medical Sciences, Tehran, Iran; 7 Iran University of Medical Sciences Colorectal Research Center Tehran Iran Colorectal Research Center, Iran University of Medical Sciences, Tehran, Iran; 8 University of Mohaghegh Ardabili Faculty of Science Department of Biology Ardabil Iran Department of Biology, Faculty of Science, University of Mohaghegh Ardabili, Ardabil, Iran; 9 Semnan University of Medical Sciences Internal Medicine Department Semnan Iran Internal Medicine Department, Semnan University of Medical Sciences, Semnan, Iran; 10 Qom University of Medical Sciences School of Medicine Department of Physiology and Pharmacology Qom Iran Department of Physiology and Pharmacology, School of Medicine, Qom University of Medical Sciences, Qom, Iran

**Keywords:** Gene, IGFBP3, insulin, NAFLD, variant

## Abstract

**Objective:**

Nonalcoholic fatty liver disease (NAFLD) is a chronic liver disease and a growing global epidemic. In NAFLD, liver fat surpasses 5% of hepatocytes without the secondary causes of lipid accumulation or excessive alcohol consumption. Given the link between NAFLD and insulin resistance, the possible association between the rs2854744 (−202 G>T) promoter polymorphism of insulin-like growth factor binding protein 3 (*IGFBP3*) gene and NAFLD was investigated in this study.

**Materials and methods:**

In this genetic case-control association study, the *IGFBP3* rs2854744 genotypes of 315 unrelated individuals, including 156 patients with biopsy-proven NAFLD and 159 controls, were determined using polymerase chain reaction/restriction fragment length polymorphism analyses.

**Results:**

The “GT+TT” genotype of the *IGFBP3* rs2854744 polymorphism, compared with the “GG” genotype, was associated with a 2.7-fold increased risk of NAFLD after adjustment for confounding factors (P = 0.009; odds ratio [OR] = 2.71; 95% confidence interval [CI] = 1.19-3.18). Additionally, the *IGFBP3* rs2854744 “T” allele, in comparison with the “G” allele, was significantly overrepresented in NAFLD patients than the controls (P = 0.008; OR = 1.85; 95%CI = 1.23-2.94).

**Conclusion:**

Our findings first indicated that the *IGFBP3* rs2854744 “GT+TT” genotype is a marker of increased NAFLD susceptibility; however, it needs to be supported by further investigations in other populations.

## INTRODUCTION

As the most common cause of chronic liver disease, nonalcoholic fatty liver disease (NAFLD) is a multifactorial metabolic disorder in which liver fat exceeds 5% of hepatocytes in the absence of the secondary causes of hepatic fat aggregation or excessive alcohol consumption. NAFLD, an emerging issue in global health, encompasses a range of diseases from the nonalcoholic fatty liver to nonalcoholic steatohepatitis (NASH) and fibrosis. Although the very high prevalence of NAFLD (approximately a quarter of adults worldwide) has placed a heavy burden on the healthcare system, its etiology is still unresolved (1). Nonetheless, prior literature has shown that NAFLD is directly linked to abnormal glucose tolerance (2), circulating insulin levels (3), insulin resistance (IR) (4), type 2 diabetes (T2D) (5), and obesity (6). The severity of IR is increased as NAFLD progresses from simple steatosis to NASH (7). IR influences the elevation rate of circulating liver enzymes in NAFLD as well; the rate is lower in NAFLD patients without IR than those with IR (8).

The insulin-like growth factor (IGF) system, which may participate in the development and progression of NAFLD, consists of two growth factors (IGF1 and IGF2), two cell-surface receptors (IGF1R and IGF2R), and six IGF binding proteins (IGFBP1 to IGFBP6). The IGF axis and insulin axis are biologically interconnected. IGF1 and IGFBP3 are implicated in the insulin signaling pathway, and IGF1 has significant homology with insulin and is similar in function to it. Given its diverse biological functions, any defects in IGF1 may lead to IR, obesity, and inflammation which are involved in NAFLD etiology. On the other hand, ~90% of IGF1 in the blood is bound to IGFBP3 – the major circulating subtype of IGFBP – which is almost exclusively produced by Kupffer cells in the liver. Human IGFBP3, the product of the *IGFBP3* gene, is a cysteine-rich polypeptide with a molecular mass of 28.7 kDa and comprises 264 amino acids. IGFBP3 controls IGF1 activities by inhibiting its bioavailability to the target tissues (9). Previous reports have also demonstrated that NAFLD patients have lower levels of serum IGF1 and IGF1 mRNA as compared to controls (10–12). Interestingly, patients with NAFLD (10) and NASH (13) have higher circulating IGFBP3 levels as well. Furthermore, IGFBP3 promotes IR (14), and its level is positively associated with the body mass index (BMI) (14). Finally, significant associations between susceptibility to NAFLD and some variants in the insulin pathway-related genes, including *IGF1*, have been found in some studies (15–20). Therefore, the present study was designed to investigate the possible contribution of the *IGFBP3* gene rs2854744 (−202 G>T) polymorphism to NAFLD. The inclusion criteria for selecting this single-nucleotide polymorphism (SNP) were its high degree of heterozygosity, high usage frequency in prior genetic studies, and location in the gene (promoter).

## MATERIALS AND METHODS

### Study population

Overall, 315 Iranian and genetically unrelated individuals [cases with biopsy-proven NAFLD (n = 156, age range of 30-87 years) and controls (n = 159, age range of 31-82 years)] were enrolled in the present retrospective case-control study. All the participants were informed of the aims of the study, and consent was obtained from them. The cases were selected from patients who were eligible. They were recruited based on clinical features, sonographic findings, and laboratory data, including the biochemical and pathological ones. The controls were recruited from eligible medical students and the Institute staff (Shahid Beheshti University of Medical Sciences). In this study, the recruitment of the NAFLD patients and the controls, the gathering of clinical, biochemical, and histopathological data, and blood sampling for genetic tests were all performed between April 2010 and August 2013. Data were collected using self-administered questionnaires. Considering that in this study, liver biopsy was used as the gold standard method for the diagnosis of NAFLD, multicenter collaborative research was conducted because of difficulties in recruiting enough NAFLD patients. However, most of the work was performed in the Research Institute for Gastroenterology and Liver Diseases, Shahid Beheshti University of Medical Sciences, Tehran, Iran. Several eligibility criteria were taken into account for selecting the cases and controls. The diagnosis of NAFLD was based on clinical features, sonographic findings, and laboratory data. To choose the cases, subjects were enrolled after the diagnosis of fatty liver defined by ultrasonographic evidence of fatty liver and high serum levels of liver enzymes, including aspartate aminotransferase (AST), alanine aminotransferase (ALT), and gamma-glutamyl transferase (GGT). Another criterion was excluding patients with other causes of liver disease, including those caused by alcohol abuse (ethanol intake per week of more than 70 g for women or 140 g for men), viral hepatitis, Wilson's disease, alpha-1 antitrypsin deficiency, or use of drugs likely to induce NAFLD. The last criterion was the confirmation of liver biopsy consistent with NAFLD by a seasoned pathologist who was blinded to the clinical and laboratory data of patients and analyzed the liver biopsy samples using Brunt's criteria. Grading of steatosis and necroinflammation was from 0 to 3, and staging of fibrosis was from 0 to 4. To select the controls, participants were excluded from the study if they had evidence of a fatty liver on abdominal ultrasonography, viral hepatitis infection on the blood test, and elevated liver enzymes on the blood test and were alcoholic or on regular medications examined by the questionnaire. The controls were of the same geographical origin as the cases. The body weight and height of each individual were obtained, and the formula for the calculation of BMI was weight in kilograms (kg) divided by height in square meters (21,22). This study followed the principles of the Declaration of Helsinki and received the approval of the Ethics Committee of the Research Institute for Gastroenterology and Liver Diseases, Shahid Beheshti University of Medical Sciences, Tehran, Iran.

### Genotyping

Blood samples were collected in ethylenediaminetetraacetic acid vials, and genomic DNA was purified from the white blood cells using phenol-chloroform extraction and ethanol precipitation protocol and then stored at −20 °C until use. The genotypes of the *IGFBP3* gene rs2854744 polymorphism were determined using the polymerase chain reaction (PCR)/restriction fragment length polymorphism method. Genotyping was performed without the knowledge of the case or control status of the participants by different laboratory personnel. The *IGFBP3* gene rs2854744 polymorphism was evaluated using 5’-CCGAGAGCGGAAGG GGTAAG-3’ and 5’-CCCGTGCTTCGCCCTGAGCA-3’ as forward and reverse primers, respectively (23). The PCR was performed with an initial denaturation at 95 °C for 10 min, followed by 38 cycles of denaturation at 95 °C for 40 s, annealing at 63 °C for 35 s, and extension at 72 °C for 45 s. The final extension was at 72 °C for 7 min. PCR products were separated by 2.5% agarose gels after digestion with the restriction enzyme of NsbI (Fermentas, St. Leon-Rot, Germany) in a water bath at 37 °C overnight. Then, the RFL fragments were stained with ethidium bromide (0.5 μg/mL) and visualized with a UV transilluminator (24). The restriction profiles and the presence (“”G” allele) or absence (“T” allele) of the NsbI determined the genotypes of the *IGFBP3* gene rs2854744 (G/T) polymorphism for each individual. NsbI digestion demonstrated genotypes denoted TT (246 bp), TG (246, 166, and 80 bp), or GG (166 and 80 bp). To ensure the genotyping results, 20% of all the samples was genotyped twice by different laboratory personnel, and the reproducibility was 100%.

### Statistical analyses

The SPSS software package for Windows (version 25.0, SPSS Inc. Chicago, IL, USA) was used to perform statistical analyses. The demographic, anthropometric, clinical, and biochemical features of the patients with NAFLD were compared with those found in the controls by Student's unpaired t-test or Chi-square (χ^2^) test as appropriate. Hardy-Weinberg equilibrium (HWE) for the *IGFBP3* rs2854744 variant among the case and control groups was also separately analyzed by using the χ^2^ test, comparing the observed genotype frequencies with the expected ones. The mentioned test was also applied to assess differences in allele frequencies between the NAFLD and control groups. Moreover, logistic regression analysis was employed to appraise the association between the genotype frequencies and NAFLD and to adjust confounding factors. The odds ratios (OR) and their respective 95% confidence intervals (95% CI) were used as the measure of strength for the associations between the *IGFBP3* rs2854744 genotypes and alleles and the risk of NAFLD. A P < 0.01 was considered a statistically significant difference.

## RESULTS

Table 1 provides the demographic, anthropometric, clinical, and biochemical characteristics of the cases with NAFLD and controls. In general, the patients with NAFLD had a higher age (P < 0.001), BMI (P < 0.001), male percentage (P < 0.001), and smoker percentage (P = 0.009) than the controls. Systolic blood pressure, diastolic blood pressure, and circulating levels of AST, ALT, and GGT were also significantly different between the case and control groups; more precisely, they were higher in the case group (P < 0.001).

**Table 1:** Demographic, anthropometric, biochemical, and clinical data of nonalcoholic fatty liver disease (NAFLD) and control groups^a^

Characteristics		Controls (n = 159)	NAFLD (n = 156)	P-value
Age (years)		28.8 (7.3)	38.5 (8.7)	<0.001
Body mass index (kg/m^2^)		22.9 (3.1)	29.7 (5.5)	<0.001
Sex				
	Male	82 (51.6)	115 (73.7)	
	Female	77 (48.4)	41 (26.3)	<0.001
Smoking history				
	No	144 (90.6)	115 (73.7)	
	Former	10 (6.3)	21 (13.5)	
	Current	5 (3.1)	20 (12.8)	0.009
Systolic blood pressure (mmHg)		113.9 (13.1)	124.0 (15.4)	<0.001
Diastolic blood pressure (mmHg)		69.9 (8.7)	75.6 (9.8)	<0.001
Aspartate aminotransferase (IU/L)		19.2 (7.1)	39.9 (17.4)	<0.001
Alanine aminotransferase (IU/L)		19.1 (10.5)	71.8 (40.5)	<0.001
Gamma glutamyl transferase (IU/L)		18.2 (8.7)	59.0 (32.1)	<0.001
Steatosis				
	Grade 0	-		
	Grade 1	41 (26.3)		
	Grade 2	84 (53.8)		
	Grade 3	31 (19.9)		
Necroinflammation				
	Grade 0	49 (31.4)		
	Grade 1	58 (37.2)		
	Grade 2	47 (30.1)		
	Grade 3	2 (1.3)		
Fibrosis				
	Stage 0	89 (57.1)		
	Stage 1	60 (38.5)		
	Stage 2	7 (4.4)		
	Stage 3	-		
	Stage 4	-		

a Variables presented as mean (SD) or number (%).

The distribution of genotypes and alleles of the *IGFBP3* gene rs2854744 polymorphism in patients with NAFLD and the controls is presented in Table 2. No deviation from HWE was observed for the *IGFBP3* gene rs2854744 variant in both case and control populations (P > 0.01). The analysis of the *IGFBP3* rs2854744 variant revealed a significant difference between the cases and controls. The “GT+TT” genotype of the *IGFBP3* rs2854744, compared with the “GG” genotype, was associated with a 2.7-fold increased risk of NAFLD after adjustment for confounding factors (P = 0.009, OR = 2.71; 95%CI = 1.19-3.18). In addition, the *IGFBP3* rs2854744 “T” allele was significantly overrepresented in NAFLD patients than the controls (P = 0.008; OR = 1.85; 95%CI = 1.23-2.94).

**Table 2:** Distribution of insulin like growth factor binding protein 3 (IGFBP3) gene rs2854744 variant in nonalcoholic fatty liver disease (NAFLD) and control groups^a^

Gene (SNP)	Controls (n = 159)	NAFLD (n = 156)	OR (95% CI) P-value^b^
*IGFBP3* (rs2854744)			
Genotype-wise comparison			
GG	82 (51.6)	50 (32.1)	1.0 (reference)
GT	55 (34.6)	71 (45.5)	1.56 (0.69-2.71) 0.306
TT	22 (13.8)	35 (22.4)	1.82 (0.78-2.66) 0.082
GT and TT	77 (48.4)	106 (67.9)	2.71 (1.19-3.18) 0.009
TT versus others	22 (13.8)	35 (22.4)	1.67 (0.84-2.85) 0.133
Allele-wise comparison			
G	219 (68.9)	171 (54.8)	1.0 (reference)
T	99 (31.1)	141 (45.2)	1.85 (1.23-2.94) 0.008

a Variables presented as number (%).

b Adjusted for age, body mass index (BMI), sex, smoking status, systolic blood pressure (SBP), and diastolic blood pressure (DBP) in genotype-wise comparisons.

## DISCUSSION

This study first investigated the possible association of the *IGFBP3* gene rs2854744 polymorphism with the risk of NAFLD. The *IGFBP3* rs2854744 “GT+TT” genotype, in comparison to the “GG” genotype, increased the risk of NAFLD more than two-fold. Moreover, the *IGFBP3* rs2854744 “T” allele was more frequent in cases with NAFLD.

The complex interaction between many genetic and non-genetic factors can dictate the presence and severity of complex diseases such as NAFLD, which has recently become a worldwide epidemic. Finding the potential genes implicated in the etiology and progression of complex diseases is really difficult regarding their moderately small individual effects. Examining the SNPs of these genes is a standard method to detect susceptibility genes. Nevertheless, it is not easy to establish whether an SNP is pathogenic or not, and unfortunately, contradictory results are not rare in genetic association studies. Epidemiological studies demonstrate that these inconsistencies can be ascribed to variations in the genetic background of different racial and ethnic groups, as well as differences in diet, lifestyle, genotyped markers, statistical methods, or even disease definition; an example of this is the procedure used for NAFLD diagnosis (25–29). Prior reports have indicated that NAFLD is linked to other metabolic disorders such as IR, T2D, obesity, and dyslipidemia. Hence, they presumably have common pathogenic mechanisms and genetic contributors. Ethnic variations in NAFLD prevalence and familial clustering represent that NAFLD has a genetic component. Genes involved in glucose metabolism, IR, fatty acid metabolism, obesity, oxidative stress, and inflammation are among the candidate genes for NAFLD. IR speeds up the flow of free fatty acids from adipocytes into the liver (4). Genetic factors can alter the risk of NAFLD, and the genes of insulin and IGF1 axes may play a crucial role in NAFLD pathogenesis. Given that IGFBP3 as the inhibitor of IGF1 bioavailability and activity has a vast array of biological functions and is involved in IR, obesity, inflammation, and oxidative stress, it seems reasonable to assume that its gene (IGFBP3) may contribute to the development and progression of NAFLD. Of note, genetic determinants such as SNPs account for approximately 40%-60% of the variability in the circulating levels of IGF1 and IGFBP3 (30).

The *IGFBP3* gene is mapped to 7p14-p12 and contains five exons. This polymorphic gene is highly conserved among species (31). In the present investigation, there was a significant association between NAFLD and the rs2854744 polymorphism located in the promoter of the *IGFBP3* gene. The “GT+TT” genotype of the *IGFBP3* rs2854744 variant, in comparison to the “GG” genotype, was a risk factor for NAFLD. The “T” allele of the *IGFBP3* rs2854744 variant, compared with the “G” allele, occurred more frequently in NAFLD patients as well. Gene expression and protein function may be affected by alterations in the promoter sequence (32). Actually, the highly functional polymorphism of rs2854744 in the promoter region of the *IGFBP3* gene is situated 202 bp upstream of the transcription start site. This SNP is strongly associated with the promotor activity of the *IGFBP3* gene and determines more than half of the changes in the circulating IGFBP3 level. The promoter activity of the *IGFBP3* rs2854744 “T” allele, compared to the “G” allele, is higher, and it is supposed that the “T” allele is associated with a higher serum IGFBP3 level. The lowest and highest concentrations of IGFBP3 are observed in individuals with the “GG” and “TT” genotypes, respectively (31,33–35). Compared with the “G” allele, the “T” allele is also associated with decreased levels of IGF1 and IGF-I/IGFBP-3 ratio, as well as increased levels of low-density lipoprotein cholesterol (31,34,36,37). Therefore, the hypothesis is that a rise in IGFBP3 levels in NAFLD patients with the “GT+TT” genotype may result in a reduction in the bioavailability of IGF1 as a hepatoprotective factor at tissue levels. Interestingly, the expression of the IGF1 receptor in the liver of patients with NAFLD is also increased, which probably acts as a compensatory mechanism to maintain the beneficial effects of IGF1 (38). Accordingly, our finding is consistent with the above-mentioned notions, indicating that the “GT+TT” genotype appears to be a risk factor for NAFLD susceptibility.

There is more evidence that supports the hypothesis that IGFBP3 may play a role in NAFLD. The *IGFBP3* gene polymorphisms are associated with circulating levels of triglyceride and high-density lipoprotein cholesterol as well (34). IGFBP3 is involved in glucose homeostasis and promotes IR, and its level is positively related to BMI (14). More interestingly, the overexpressing of IGFBP3 in transgenic mice leads to IR (39), probably due to decreased IGF1 bioavailability. Other studies represented that in the general population, adults with a higher IGF1/IGFBP3 ratio have a lower NAFLD risk (40), and NAFLD patients have a lower IGF1/IGFBP3 ratio (41). Moreover, IGF1 is negatively associated with liver enzymes, IR, and oxidative stress (14,42). Consistently, IGF1 has anti-inflammatory effects on hepatic cells (43) and is negatively associated with the degree of inflammation (44). On the other hand, inflammatory cytokines reduce IGF1 expression (43). The IGF1/IGFBP3 ratio is the major predictor of liver inflammation in children with NAFLD (44). A low IGF1/IGFBP3 ratio also acts as a marker of advanced NAFLD (41). Moreover, it has been demonstrated that treatment with exogenous IGF1 and IGFBP3 can prevent the release of pro-inflammatory cytokines, namely, interleukin-1β and tumor necrosis factor-α (45). Hepatic steatosis and hepatic fibrosis are negatively associated with IGF1 (10,11,14,44,41) and IGF1/IGFBP3 ratio (41,46), while positively associated with IGFBP3 (47,48). Finally, higher IGFBP3 levels in NAFLD patients may reduce the bioavailability of IGF1 in the liver tissue, including the overexpression of microRNA-190b (miR-190b) in patients with NAFLD which suppresses IGF1 expression and induces lipid accumulation and IR. MiRNAs are non-coding and single-stranded RNA molecules that contain 22-25 nucleotides which act in the post-transcriptional regulation of gene expression (49,50). Therefore, a growing body of research suggests that IGFBP3 and its gene (*IGFBP3*) participate in the etiology of NAFLD. Nonetheless, the exact mechanism through which the *IGFBP3* rs2854744 polymorphism may influence the function of visfatin and NAFLD pathogenesis is unclear.

This study had some limitations that might have affected its findings. First, the sample size of our study was modest because of using liver biopsy, and for this reason, performing sub-analyses was unreasonable. Second, owing to budget limitations, we were unable to measure the IR (HOMA-IR) index. Third, considering the genotyping of only one polymorphism in the *IGFBP3* gene, the coverage of the gene was incomplete. Despite these limitations, this study had some strengths which should be kept in mind as well. In the current case-control study, which had a good design, liver biopsy was employed as the gold standard method to confirm NAFLD diagnosis. Additionally, to the best of our knowledge, our report is the first one that has investigated the association between *IGFBP3* gene variants and the risk of NAFLD. Eventually, in line with previou reports, novel and interesting results were found in this research.

In conclusion, our findings first demonstrated that the *IGFBP3* gene may play a role in the pathogenesis of NAFLD; the *IGFBP3* rs2854744 “GT+TT” genotype had a 2.7-fold increased risk of NAFLD compared with its “GG” genotype counterpart. Interestingly enough, this observation is pertinent from a theoretical viewpoint; nonetheless, further studies with larger samples of different populations are required to elucidate the participation of *IGFBP3* gene variants in NAFLD susceptibility.
